# Echo-ODE: A dynamics modeling network with neural ODE for temporally consistent segmentation of video echocardiograms

**DOI:** 10.3389/fphys.2025.1629121

**Published:** 2025-08-18

**Authors:** Wenliang Lu, Yuan Wang, Wenli Dai, Yingnan Wu, Hao Xu, Dexing Kong

**Affiliations:** ^1^ School of Mathematical Sciences, Zhejiang University, Hangzhou, Zhejiang, China; ^2^ Department of Mathematics, Zhejiang Sci-Tech University, Hangzhou, Zhejiang, China; ^3^ Department of Ultrasound Medicine, Litao Sun Cancer Center, Zhejiang Provincial People’s Hospital (Affiliated People’s Hospital), Hangzhou Medical College, Hangzhou, Zhejiang, China; ^4^ College of Mathematical Medicine, Zhejiang Normal University, Jinhua, Zhejiang, China; ^5^ Cardiovascular Research Group, Puyang Institute of Big Data and Artificial Intelligence, Puyang, Henan, China

**Keywords:** echocardiogram, cardiac segmentation, temporal consistency, physical dynamics representation, neural ordinary differential equations

## Abstract

**Introduction:**

Segmentation of echocardiograms plays a crucial role in clinical diagnosis. Beyond accuracy, a major challenge of video echocardiogram analysis is the temporal consistency of consecutive frames. Stable and consistent segmentation of cardiac structures is essential for a reliable fully automatic echocardiogram interpretation.

**Methods:**

We propose a novel framework Echo-ODE, where the heart is regarded as a dynamical system, and we model the representation of dynamics by neural ordinary differential equations. Echo-ODE learns the spatio-temporal relationships of the input video and output continuous and consistent predictions.

**Results:**

Experiments conducted on the Echo-Dynamic, the CAMUS and our private dataset demonstrate that Echo-ODE achieves comparable accuracy but significantly better temporal stability and consistency in video segmentation than previous mainstream CNN models. More accurate phase detection and robustness to arrhythmia also underscore the superiority of our proposed model.

**Discussion:**

Echo-ODE addresses the critical need for temporal coherence in clinical video analysis. This framework establishes a versatile backbone extendable beyond segmentation tasks. Its ability to model cardiac dynamics demonstrates great potential for enabling reliable, fully automated video echocardiogram interpretation. The code is publicly available at https://github.com/luwenlianglu/EchoODE.

## 1 Introduction

Accurate assessment of cardiac structure and function is crucial to clinical diagnosis and guide optimal treatment options for patients (Alsharqi et al., 2018). Echocardiography is one of the most widely used imaging modalities that allows real-time imaging and can detect various abnormalities. A key aspect of echocardiography is the assessment and quantification of cardiac chamber size and function, particularly the left ventricle (LV). Quantification of left ventricular ejection fraction (LVEF), for example, is the central measure of LV systolic function (Loehr et al., 2008; Huang et al., 2017), and it is calculated by the fraction of LV volume of the end-systolic (ES) and end-diastolic (ED). Currently, sonographers typically determine the cardiac phase of an echocardiogram visually. They track ED frames and ES frames through consecutive cardiac cycles and then manually delineate the chamber boundary to calculate LVEF and other functional metrics. This process is time-consuming, tedious and prone to poor repeatability (Koh et al., 2017). Furthermore, LVEF assessment is recommended to average the results of several consecutive cardiac cycles if variation is identified. However, in practice, it is often evaluated from tracings of just one representative beat (Lang et al., 2015), leading to high variance and limited precision due to inter-observer variation (Malm et al., 2004; Farsalinos et al., 2015). While automatic segmentation of video echocardiograms can solve the problem, temporally inaccurate or inconsistent segmentation can lead to incorrect phase detection, resulting in unreliable LVEF calculations.

Early attempts to algorithmically assess cardiac function applied deep learning models to manually curated still images on ED frames and ES frames (Zhang et al., 2018; Moradi et al., 2019; Moradi et al., 2019; Liu et al., 2021; Guo et al., 2021; Su et al., 2024; Akbari et al., 2024), or segmenting multiple planes in one model Hu et al. (2025). Some subsequent works extended these image-level models to echocardiogram videos, analyzing them frame-by-frame and plotting the curve of left ventricular volume over time. The peak and valley points of the curve were recognized as ED frames and ES frames (Ouyang et al., 2020; Duffy et al., 2022). Although these models allowed fully automated cardiac function assessment, they ignored the temporal dependencies between video frames. Later models sought to combine spatial and temporal information (Lane et al., 2021; Wu et al., 2022; Zeng et al., 2023; Ta et al., 2024; Hasan et al., 2025). These models processed a few consecutive frames as input and predicted the label for the last frame. Models in this category were still considered image-level models, as only a single frame prediction could be derived from each computation. The approaches mentioned above either applied image-level nets to each frame or used consecutive frames as input but only output a one-frame prediction, and none of them guaranteed temporal consistency. Therefore, it is necessary to promote a temporally stable and consistent segmentation algorithm for video echocardiograms.

Inspired by reference (Özçelik and Altan, 2023) to deal with non-linear dynamics, we sought a new framework capable of overcoming these challenges and specifically designed to handle such a unique system. Recall that a dynamical system consists of an abstract phase or state space, where the coordinates describe the system’s state at any given moment, and a dynamical rule that specifies the future evolution of the system based solely on the present values of its state variables. Intuitively, the heart clearly represents a biological dynamical system. The processes of diastole and systole over time constitute the state space. The heart beats regularly controled by the cardiac conduction system, allowing us to learn the dynamic rule and predict future states. This dynamic property still holds when applied to video echocardiograms, which represent a sectional view or subspace of the entire heart space. Mathematically, a dynamical system is described by the initial value problem of a differential equation, which inspired us to model the echocardiogram using neural ordinary differential equations (NODE). Notably, many differential equation solvers include a scheme known as dense output, which generates high-order interpolations by reusing internal computations from the steps without requiring additional computational resources or time (Shampine and Jay, 2015). The advantages of modeling echocardiograms with differential equations are as follows: (1) The heart is treated as a dynamical system, making the model more interpretable; (2) The input series does not need to have a constant time duration, unlike the requirement in RNN models; (3) The outputs of NODE are dense and continuous in the time dimension, allowing us to test our model on real videos with non-adjacent input frames. Intermittent sampling is possible, and internal predictions can be generated through dense outputs, enhancing computational efficiency; (4) The continuous outputs of NODE make it feasible to perform some video generation tasks, such as video extrapolation and interpolation on sparsely sampled echocardiograms.

## 2 Materials and methods

### 2.1 Neural ordinary differential equations

NODEs (Chen et al., 2018) represent a family of parameterized algorithms designed to model the evolution over time of a system with state ξ(t) at an arbitrary time t . These systems are governed by continuous-time dynamics that satisfy a Cauchy (or initial value) problem:

$$\left\{\begin{array}{cc} \frac{\partial \xi }{\partial t}\left(t\right)\; & =f\left(\xi \left(t\right),t\right),\\ \xi \left(t_{0}\right)\; & =\xi_{0}. \end{array}\right.$$

By approximating the differential with an estimator $f_{\theta }≃f$ parameterized by θ , such as a neural network, these methods enable learning of such dynamics (or trajectories) from relevant data. With this formalization, the state ξ(t) of the system is defined at all times, and can be computed at any desired time using a numerical ODESolver

$$\left(\xi \left(t_{0}\right),\xi \left(t_{1}\right),\ldots ,\xi \left(t_{n}\right)\right)=ODESolver\left(f_{\theta },\xi_{0},\left(t_{0},t_{1},\ldots ,t_{n}\right)\right).$$

For any single arbitrary time value $t_{i}$ , a call to ODESolver computes a numerical approximation of the integral of dynamics from the initial time value $t_{0}$ to $t_{i}$ .

$$\xi \left(t_{i}\right)=ODESolver\left(f_{\theta },\xi_{0},\left(t_{0},t_{i}\right)\right)=\xi_{0}+\int_{t_{0}}^{t_{i}}f_{\theta }\left(\xi \left(s\right),s\right)ds.$$

A plethora of algorithms for numerical integration of differential equations can be found in the existing literature. The optimization of neural ODEs is carried out within the framework of adjoint sensitivity (Pontryagin, 1985).

Inspired by the application of NODEs to continuous time series modeling, the latent ODE (Rubanova et al., 2019) equipped with an ODE-RNN was proposed to handle irregularly sampled time series data. Ma et al. (2022) introduced CortexODE, which leveraged NODEs to learn a diffeomorphic flow for the reconstruction of the cortical surface, ensuring that the reconstruction preserved the topology. ODE2VAE (Yildiz et al., 2019) aimed to decompose latent representations into position and momentum to generate low-resolution image sequences. Park et al. (2021) proposed Vid-ODE, a network for continuous-time video generation.

### 2.2 Notation and overview

#### 2.2.1 Notation

We denote $X_{\tau }≔\left\{X_{t_{1}},X_{t_{2}},\ldots ,X_{t_{n}}\right\}$ as a sequence of input video frames of length n, where each $X_{t_{i}}\in R^{h\times w\times c}$ is a 2D image of size h×w with c channels sampled at either regularly or irregularly spaced timesteps $\tau ≔\left\{t_{1},t_{2},\ldots ,t_{n}\right\}$ , with $0<t_{1}<t_{2}<\ldots <t_{n}$ . Given an input video $X_{\tau }$ and a specific task T for another set of time steps $S≔\left\{s_{1},s_{2},\ldots ,s_{m}\right\}$ , our goal is to generate the predictions $T_{S}≔\left\{T_{s_{1}},T_{s_{2}},\ldots ,T_{s_{m}}\right\}$ at these timesteps. S needs not to be the same as τ and can be any countable subset of the interval $\left[0,t_{n}\right]$ . The origin is defined as $t_{0}=s_{0}=0$ .

#### 2.2.2 Overview

**FIGURE 1:**
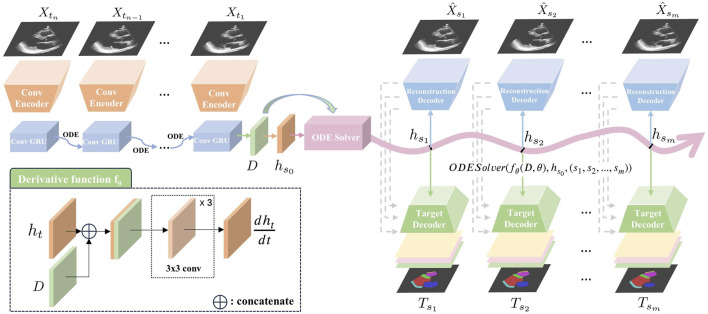
Overview of our proposed Echo-ODE. A convolutional encoder embedded the input video clip into the hidden states. It was followed by the latent ODE, ODESolver and convolutional decoders to generated the target prediction. $X_{t_{n}}$ : input images. ${\hat{X}}_{s_{n}}$ : reconstructed image. $T_{s_{n}}$ : target output, generated by adjusting the decoder head according to different tasks.

An overview of our proposed Echo-ODE framework is shown in Figure 1. Echo-ODE adopts an Encoder-Decoder structure. First, a convolutional encoder is applied to reduce the dimension and capture the spatial features of all frames in the input clip with shared parameters, it is followed by a GRU-ODE (Latent ODE) block to capture the temporal dependencies and learn the overall infomation and initial state of the video (top left of Figure 1, details described in Section 2.3). These outputs are then fed into an ODESolver to obtain continuous hidden states at arbitrary time steps. These hidden states are used to generate the desired predictions for a certain task by a target decoder (bottom right of Figure 1). To enable the model to learn the essential representations of the video, this paper adds a self-supervised video reconstruction block (top right of Figure 1), which is also a shared parameter convolutional network. A skip connection from the reconstruction decoder to the target decoder enhances the target decoder’s ability to capture high-resolutional semantic features (details described in Section 2.4).

### 2.3 Encoder

Given the input $\left\{X_{t_{i}}\right\}$ , a convolutional encoder E (top left of Figure 1) is applied to learn the spatial feature and embed the video frames into hidden states $\left\{{\hat{h}}_{t_{i}}\right\}$ . The encoder consists of four pairs of convolutional layers with max-pooling between adjacent convolutional pairs, resulting in four different resolutions. The number of 3×3 convolutional kernels is (32, 32), (64, 64), (128, 128), (256, 256) in each pair. The encoder shares the same parameters across different frames of the clip. Extracted features can be expressed as:

$${\hat{h}}_{t_{i}}=E\left(X_{t_{i}}\right).$$

It is followed by a latent ODE (Rubanova et al., 2019) to capture the temporal relationships and learn the overall dynamic information D . An initial state $h_{s_{0}}$ is derived from D by a convolutional layer. The latent ODE is a combination of neural ODE (Chen et al., 2018) and ConvGRU (Ballas et al., 2015). While NODE can successfully model temporal dynamics at irregular time steps, it cannot directly process media input. On the other hand, RNN models struggle with irregular time input. To address this issue, the latent ODE is designed to capture the temporal dependencies with irregular timestep media input.

To obtain a better initial state $h_{s_{0}}$ that will be as one of the inputs of ODESolver , we apply the latent ODE backward-in-time as suggested by the authors of Rubanova et al. (2019), i.e., from the last frame of the input clip to the first one. In our case, the latent ODE can be formulated as:

$$\begin{array}{cc} h_{t_{i-1}}^{-} & =ODESolver\left(f,h_{t_{i}},\left(t_{i},t_{i-1}\right)\right),\\ h_{t_{i-1}} & =ConvGRUCell\left(h_{t_{i-1}}^{-},E\left(X_{t_{i-1}}\right)\right), \end{array}$$

where i=n,n−1,…,1 . The ODE solver calculates the next hidden state $h_{t_{i-1}}^{-}$ by integration $\frac{dh_{t_{i}}}{dt}$ , which is approximated by a neural network f . Then $h_{t_{i-1}}^{-}$ is updated with an encountered media input $E\left(X_{t_{i-1}}\right)$ at the current time using a ConvGRU cell to produce $h_{t_{i}}$ . The initial hidden state $h_{t_{n+1}}=h_{t_{n}+\epsilon }$ is set to zeros, with ϵ set to 0.01. The obtained $h_{s_{0}}$ serves as the initial value of the NODE decoder, and the overall dynamic information D is integrated into the neural derivative function.

### 2.4 Decoder

#### 2.4.1 Baseline

The decoder uses NODE to generate a sequence of frames at the target timesteps $\left\{s_{1},s_{2},\ldots ,s_{m}\right\}$ based on the latent representation of the initial state $h_{s_{0}}$ and the derivative function $f_{\theta }\left(D,\theta \right)$ . The newly generated hidden state at any time $s_{i}$ can be presented as:

$$h\left(s_{i}\right)=h_{s_{0}}+\int_{s_{0}}^{s_{i}}f_{\theta }\left(D,\theta \right)ds,\;i=1,2,\ldots ,m.$$

This process can be solved by any ODESolver .

We observe that the derivative function $f_{\theta }$ should be unique for each video clip because every heart follows its own dynamic pattern. To the best of our knowledge, models that apply NODE to time series up to now only learn the initial state, and the derivative function is fixed once the training process is complete. These models typically perform training and testing on the same time series to accomplish tasks such as interpolation and extrapolation. In other words, these models fine-tune the parameters for each sample individually, which is clearly not suitable for our case. The reason for these limitations is that their derivative functions are fixed and only receive the state variable as input. To address this limitation, we integrate the heart dynamic information learned from each clip into the derivative function, as shown in the bottom left of Figure 1. For any time t , the function concatenates the hidden state $h_{t}$ and the dynamic information D , which is then passed through three convolutional layers to compute the derivative. This derivative function dynamically adjusts to different video inputs through D , eliminating the need for video-specific training.

Note that most ODE solvers employ a scheme known as dense output to generate high-order interpolations by reusing the internal computations from the solver steps (Shampine and Jay, 2015). This interpolation scheme often requires no additional calculations, allowing the target time steps to be any subset of time, regardless of whether the time series is regular or irregular, sparse or dense. Due to the dense output of NODE, we can obtain intermediate predictions even when the input consists of sparsely sampled frames, thereby improving computational efficiency.

#### 2.4.2 Dynamic representation learning

Theoretically, a target decoder (bottom right of Figure 1) can accomplish our task on its own, and it is provided as a baseline for our method in this work. However, video echocardiograms are typically annotated on only one or two frames in practice, making it difficult for our model to fully understand the dynamics of the heart by relying solely on sparsely annotated task labels.

To address this issue, we add a reconstruction block as a self-supervised learning strategy to capture the essential representation of the video (top right of Figure 1). The reconstruction decoder consists of a sequence of upsampling convolutional layers that reverse the encoder process and produce three-channel reconstructed images. Since the output time steps do not have to be the same as the input, this block functions as image reconstruction when the target time node coincides with one of the input time steps. Otherwise, it can be interpreted as video interpolation. Both video reconstruction and video interpolation encourage the network to gain a better understanding of the heart dynamics and help it learn the essential dynamic representation within NODE.

#### 2.4.3 Skip connections

Motivated by the strong performance of U-Net (Ronneberger et al., 2015) on medical images, where the skip connections between the contracting and expansive paths help retain high-resolution semantic features, we aim to preserve this property. However, direct skip connections from the encoder to the target decoder are not feasible due to the mismatch in time steps. As shown earlier, we achieve video interpolation during representation learning, so the expansive path for video generation can provide high-resolution semantic features when aligned with the target time steps. We introduce skip connections between the reconstruction decoder and the target decoder, allowing the learned video representation to enhance the performance of our target decoder.

#### 2.4.4 Target decoder

In this work, our primary goal is the video segmentation of intracardiac structures, so the target predictions $T_{S}$ are the segmentation masks of classes containing background and other intracardiac structures (the number and types of intracardiac structures varies among different datasets, details are described in Section 2.6). Therefore, the target decoder consists of a sequence of upsampling convolutional layers similar to the reconstruction decoder and produce a multi-channel output corresponding to the probabilities of each class. An argmax function is applied to obtain the final segmentation masks. Since our proposed model is not limited to video segmentation, the target decoder can be adapted for different tasks.

### 2.5 Loss function

We apply the commonly used loss functions to verify the validity of our proposed framework. Other loss functions are also acceptable. The reconstruction loss is the mean square error:

$$\ell_{R}=MSE\left(\left\{X_{s_{i}}\right\},\left\{{\hat{X}}_{s_{i}}\right\}\right),$$

where ${\hat{X}}_{s_{i}}$ is the reconstructed image by reconstruction decoder at $s_{i}$ corresponding to the real image $X_{s_{i}}$ . The cross entropy loss and dice loss is applied to segmentation loss:

$$\ell_{S}=CE\left(M,\hat{M}\right)+Dice\left(M,\hat{M}\right),$$

where M and $\hat{M}$ are the ground truth and predicted segmentation mask respectively. The overall loss function is

$$\ell =\ell_{S}+\lambda \cdot \ell_{R},$$

where λ is a hyper-parameter.

### 2.6 Datasets

In our experiments, we use a private dataset of PLAX videos and two public datasets, Echo-dynamic and CAMUS. The three datasets are described in detail below.

#### 2.6.1 Private dataset

The resource of our private dataset comes from the Echonet-LVH (Duffy et al., 2022), which includes 12,000 labeled PLAX echocardiogram videos and expert annotations (measurements and calculations) to provide a baseline for studying the size of the cardiac chambers and the thickness of the wall. It lacks the segmentation masks of the cardiac structures. Because some samples of Echo-LVH are not of good quality, the videos are selected on the basis of video quality, which mainly takes into account the image size and clarity, the video recording method, and whether it is a PLAX video. Eventually, 2067 videos are selected. Afterward, each video is re-labeled on one or two frames, which are either ED frames or ES frames. The new annotations add labels for the segmentation of cardiac structures, including the contour of the LV, left atrium (LA), right ventricle (RV), left ventricular posterior wall (LVPW), interventricular septum (IVS) and right ventricular anterior wall (RVAW). In addition, more measurement labels are annotated, including LVPW thickness, LV internal dimension (LVID), IVS thickness, LA internal dimension, left ventricular outflow tract posterior dimension, right ventricular anterior wall thickness. Measurement labels annotate the coordinates of the starting and ending points and the length of the line. The labels do not necessarily exist on every annotated frame because some of those structures are not wholly visible in the image. Therefore, though the source video echocardiograms of this dataset is publicly available, the annotations being performed makes it private. All these works are conducted by an expert sonographer from Zhejiang Provincial People’s Hospital (Affiliated People’s Hospital), with more than 5 years of working experience.

#### 2.6.2 EchoNet-dynamic

The EchoNet-Dynamic (Ouyang et al., 2019) includes 10,030 apical four-chamber view videos with corresponding labeled measurements, including ejection fraction, LV volume in ED and ES, and human expert tracings of the LV as an aid for studying machine learning approaches to evaluate cardiac function. It is the largest labeled medical video dataset made available publicly to researchers and provides a baseline for studying cardiac motion and chamber sizes.

#### 2.6.3 CAMUS

The CAMUS dataset (Leclerc et al., 2019) is the largest publicly available fully annotated dataset for 2D echocardiographic assessment. The purpose of this dataset is to provide all materials to the community to solve the problem of echocardiographic image segmentation and volume estimation from 2D ultrasound sequences. It contains 2D apical four chamber (4CH) and two chamber (2CH) view sequences acquired from 500 patients, and the videos are annotated frame by frame. Segmentation masks include the LV, LA, and myocardium of the left ventricle (LVM).

### 2.7 Temporal consistency metrics

Usually, the intersection over union (IoU) and dice coefficient (Dice) are used to show the performance of segmentation and to measure the region similarity of predicted and label masks. However, temporal consistency of the video segmentation is also an important aspect in video segmentation since the evolution of object shapes is an important cue for relative downstream tasks. Stable temporal segmentation contributes to the automatic analysis of the heart, such as cardiac wall motion and many downstream tasks. Therefore, we utilize two metrics to measure temporal consistency.

From the perspective of the predicted video segmentation itself, we calculate the TC proposed by Perazzi et al. (2016). This method transforms a mask $M_{t}$ in time t into polygons representing its contours $P\left(M_{t}\right)$ , then describes each point $p_{t}^{i}\in P\left(M_{t}\right)$ using the Shape Context Descriptor (SCD) (Belongie et al., 2002). The matching of two masks is presented as Dynamic Time Warping (DTW) (Rabiner and Juang, 1993) problem that minimizes the SCD distances between the matched points while preserving the order in which the points are present in the shapes. The resulting mean cost per matched point is used as a measure of the temporal stability τ of this pair of segmentation. The smaller the metric, the better. In our paper, the mean mask matching of all adjacent frames is used to measure the temporal consistency of the whole video segmentation:

$$TC=\frac{1}{NT}\overset{N}{\underset{k=1}{\sum }}\overset{T}{\underset{t=1}{\sum }}\tau \left(M_{k}^{t-1},M_{k}^{t}\right),$$

where $M_{k}^{t}$ is the predicted mask of t-th frame in k-th video.

In terms of scenarios where the ground truth of the whole video is available, we propose a more intuitive metric based on the ground truth. Assuming that the labels are consistent and the predictions are also consistent and well aligned with the labels, then the dice of any adjacent segmentation of a consistent prediction should be very close. We define temporal consistency of dice as:

$$TC_{D}=\frac{1}{NT}\overset{N}{\underset{k=1}{\sum }}\overset{T}{\underset{t=1}{\sum }}|D_{k}^{t}-D_{k}^{t-1}|,$$

where $D_{k}^{t}$ is the dice similarity of t-th frame in k-th video. theoretically, $TC_{D}$ should be close to zero if the predictions are temporal consistent.

## 3 Results

### 3.1 Implementation details

Model design and training are done in Python using the PyTorch deep learning library on two NVIDIA Tesla P40 GPUs. The private dataset is split into train, valid and test subsets in the ratio of 8:1:1. These subsets of the other two public datasets are split by the publisher. Stochastic gradient descent (SGD) optimizer with a learning rate of 0.05 is used. We train Echo-ODE for 100 epochs with a batch size of 16. Random resize, random crop, random horizontal flip, color jitter, and random Gaussian blur are used for data augmentation. For hyperparameters, λ is set to 10 by empirical grid search over a focused range {1,5,10,20} that balance the magnitude scales of the two loss components. Dopri5 is chosen as our ODESolver. The time interval for integrating the NODE is set to [0,1], i.e., $t_{n}=1$ . Therefore, the timesteps of the input τ and output S is determined by the frame sampling rate. When frames are sampled at uniform intervals as it is the case in this paper, the timenodes are also uniformly spaced within the time interval [0, 1]. All of the experiments in this paper are trained from scratch. To show the effectiveness of our model, we compare the performance of Echo-ODE with the classical image-level model UNet Ronneberger et al. (2015) and the classical RNN-based video-level model ConvLSTM Hochreiter and Schmidhuber (1997) with the same loss functions. In order to compare the ability of modeling the dynamics of echocardiograms by LSTM cells and NODE, we replace the NODE blocks in Echo-ODE by LSTM cells to capture the temporal relationship of hidden states and abbreviate this method as Echo-LSTM. UNet is trained at the image level and applied to every frame during video prediction, just as most works have done so far. The inputs of ConvLSTM are the same as those of Echo-LSTM and Echo-ODE, which sample 4 images from the video every two frames around the annotated image. In the training stage, the labeled frame is randomly ranked in one of the 4 frames. In the validation and test stages, the labeled frame is set to be fixed in the last frame for repeatability of experiments. When we need to output the prediction of a full video, Echo-ODE gets all the 7 intermediate predictions of the clip by dense outputs, and ConvLSTM is carried out 2 times with each time a frame shift of the inputs to get all the predictions. The loss convergence behavior of the training process is shown in Supplementary Material. Training and validation loss of Echo-ODE demonstrate a smooth and steady decrease, reaching a stable plateau after approximately 80 epochs without signs of overfitting or underfitting. The reconstruction loss and segmentation loss have the same magnitude after scaled by the weight parameter λ , and the synchronized convergence of both losses suggests that the reconstruction and the segmentation mutually reinforce each other during joint training.

### 3.2 Performance on private dataset

In this section, we first verify that Echo-ODE is a valid method for representing heart dynamics. Then, we compare the segmentation performance of Echo-ODE with UNet, ConvLSTM, and Echo-LSTM in three aspects: region similarity, temporal consistency, and the ability to perform phase detection.

#### 3.2.1 Dynamic representation

**FIGURE 2:**
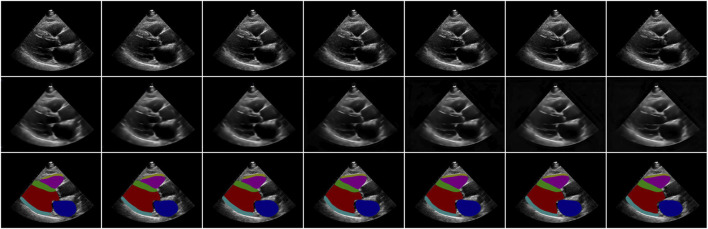
Examples of video reconstruction and segmentation by Echo-ODE. Top row: original images. Middle row: reconstructed images. Bottom row: segmentation masks.

Figure 2 shows an example of video reconstruction and segmentation by Echo-ODE. It is a single run with video interpolation. As we can see, the textures of the original images are reconstructed perfectly except for the noise, and they are more likely to be the smoothed images. This is within our expectation because there are no skip connections from the encoder path and some details are missing. Since the images are reconstructed directly from the NODE, we can draw a tentative conclusion that the NODE model is largely capable of learning certain dynamic characteristics of the video and that NODE is a reliable method to model the dynamics of the heart.

#### 3.2.2 Region similarity

The IoU and the dice are summarized in Table 1. As segmentation of LV is the most critical task in many applications, we list the dice of LV segmentation and the corresponding 95% confidence interval of these four methods in Table 2.

**TABLE 1:** Comparison of segmentation performance of six cardiac structures. Higher values indicate better performance. The best results are highlighted in bold.

Method	Region similarity (IoU/Dice)						
	LA	IVS	LVPW	LV	RV	RVAW	Mean
UNet	**0.842/0.914**	**0.788/0.882**	**0.610/0.757**	**0.867/0.928**	**0.875/0.933**	0.406/0.577	**0.731**/0.832
ConvLSTM	0.828/0.906	0.782/0.877	0.599/0.749	0.862/0.926	0.855/0.922	0.433/0.604	0.726/0.831
Echo-LSTM	0.825/0.904	0.779/0.876	0.593/0.745	0.856/0.922	0.854/0.921	0.401/0.574	0.718/0.824
Echo-ODE	0.830/0.907	0.776/0.874	0.593/0.745	0.856/0.922	0.870/0.931	**0.452/0.622**	0.730/**0.834**

**TABLE 2:** Dice and 95% confidence interval comparison of LV segmentation. The data format is Dice (confidence interval). Higher values indicate better performance. The best results are highlighted in bold.

Method	ED	ES	Overall
UNet	**0.942(0.936–0.948)**	**0.908(0.897–0.919)**	**0.928(0.921–0.935)**
ConvLSTM	0.940 (0.933–0.946)	0.905 (0.893–0.918)	0.926 (0.918–0.934)
Echo-LSTM	0.935 (0.929–0.941)	0.903 (0.891–0.915)	0.922 (0.914–0.930)
Echo-ODE	0.937 (0.931–0.943)	0.900 (0.886–0.914)	0.921 (0.914–0.928)

Tables 1, 2 show that UNet, ConvLSTM, and Echo-ODE exhibited comparable performance in terms of the overall mean dice score. UNet demonstrates the best performance across most structures, but struggles with RVAW segmentation, which has fewer training instances. The dice score for Echo-ODE is slightly lower than that of UNet and ConvLSTM. We attribute the advantages of the latter two methods to two main factors. First, UNet is an image-level model that focuses on learning the ED and ES frames. The evaluation process is also carried out on these images, so UNet performs better in its specialized domain, while the other two methods allocate some of their focus to learning segmentation on images beyond just the ED and ES frames. Furthermore, both UNet and ConvLSTM utilize skip-connections from the encoder path, which combine low-level and high-level semantic features extracted by the model, providing richer information for the final output. In contrast, the Echo-ODE model directly decodes the segmentation results from the NODE output, leading to the loss of low-level information. However, our model achieves an accuracy comparable to that of the other two methods.

Compared to ConvLSTM, Echo-LSTM loses low-level semantic features from the encoder, resulting in lower segmentation performance (mean Dice: 0.718 vs. 0.726). Surprisingly, Echo-ODE achieves superior segmentation metrics despite similar feature limitations, outperforming ConvLSTM (mean Dice: 0.730 vs. 0.726) The structures of Echo-LSTM and Echo-ODE are the same except for the bottleneck, which suggests that NODE is more suitable than LSTM for modeling cardiac dynamics. This is because ODE learns the continuous derivation of echocardiograms, whereas LSTM just learns from discrete inputs.

Another potential strength of Echo-ODE is its ability to resist missed labels. As mentioned earlier, the segmentation is not fully labeled in our private dataset, and the RVAW is the least labeled, although the structure is partially visible in some of these images. Echo-ODE can deduce RVAW segmentation benefiting from a better dynamic representation and performs far better than UNet and ConvLSTM.

#### 3.2.3 Temporal consistency

**FIGURE 3:**
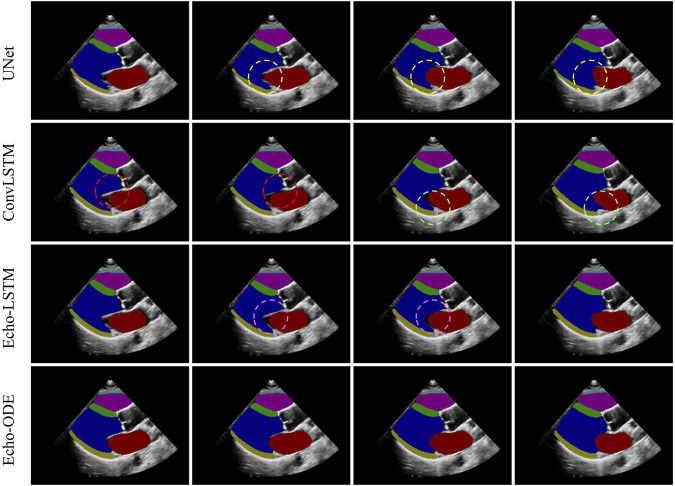
Segmentation of a clip of neighbouring frames. Dashed circles of the same color mark inconsistencies (large fluctuations) of the segmentation results between adjacent frames.

The results of TC are summarized in Table 3. Echo-ODE performs best in terms of temporal consistency in all six cardiac structures and is much more consistent than the other three methods. To provide a better visual perception, we use a continuous clip as an example and compare the segmentation results of the four methods in Figure 3. As shown, in the regions marked by dashed circles of the same color, the segmentation masks exhibit large fluctuations between adjacent frames, while Echo-ODE apparently performs better in these regions. Due to the lack of temporal context information, the predictions of UNet may be affected by image noise, resulting in large differences even when the changes between original images are visually subtle. Echo-ODE performs much better in terms of temporal consistency, which not only improves the TC by 20.28% , 21.61% and 18.68% compared to the other methods respectively, but also visually appears significantly more consistent.

**TABLE 3:** Comparison of TC of video segmentation. Lower values indicate better performance. The best results are highlighted in bold.

Method	LA	IVS	LVPW	LV	RV	RVAW	Mean
UNet	0.334	0.337	0.289	0.198	0.341	0.631	0.355
ConvLSTM	0.353	0.360	0.307	0.215	0.382	0.551	0.361
Echo-LSTM	0.372	0.332	0.284	0.210	0.288	0.604	0.348
Echo-ODE	**0.330**	**0.311**	**0.226**	**0.184**	**0.261**	**0.387**	**0.283**

#### 3.2.4 Phase detection

**FIGURE 4:**
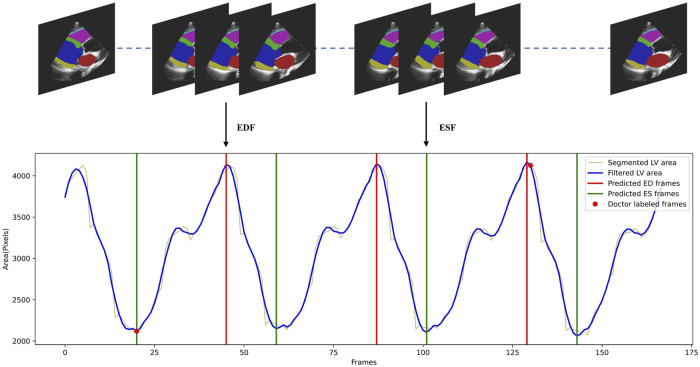
Illustration of the cardiac cycle phase detection process. The LV areas of a video along time was smoothed by Savitzky-Golay filtering algorithm, then the detected peaks and valleys of the curve were regarded as ED and ES frames.

To demonstrate that our temporally consistent model is more reliable in clinical scenarios, we test our model in downstream tasks. In a typical workflow for fully automated analysis of video echocardiograms, we first segment every frame of the video, then select the appropriate frames as ED and ES frames (phase detection) based on the segmentation results. Indicators such as the ejection fraction are then calculated to evaluate whether the heart is healthy. We conduct phase detection as an example to test whether the segmentation by Echo-ODE can effectively accomplish downstream tasks with better performance. The method follows the reference Zeng et al. (2023). All frame sequences $P_{n}$ of the LV segmentation are extracted and a curve of the left ventricular area A is plotted as a function of time. The Savitzky-Golay filtering algorithm with filter width of 13 and polynomial order of three is applied to reduce noise interference. Finally, a peak detection function is used to identify peaks and valleys as ED and ES frames. This process is shown in Figure 4, and more details can be found in reference Zeng et al. (2023). Since our dataset is annotated with ED and ES frames by experts, these frames are regarded as the ground truth. A detected peak within 10 frames of the ground truth is considered a valid prediction. The mean absolute error of phase detection is listed in Table 4.

**TABLE 4:** Errors of cardiac cycle phase detection by different methods. Lower values indicate better performance. The best results are highlighted in bold.

Method	MAE (frames)		
	ED	ES	Overall
UNet	1.71	2.47	2.13
ConvLSTM	**1.56**	2.19	1.91
Echo-LSTM	1.65	2.32	2.02
Echo-ODE	1.57	**1.98**	**1.79**

As shown in Table 4, Echo-ODE has the least detection error in both ED frames, ES frames and overall compared to the other three methods. It shows that a consistent and stable segmentation of cardiac structures can be benifical in completing the phase detection task.

#### 3.2.5 Robustness to arrhythmia

To demonstrate the robustness of our model in handling diverse cardiac conditions in clinical scenarios, we analyze the segmentation performance of patients with arrhythmia in the validation and test datasets, which include a total of 18 cases. The results are listed in Table 5. Compared to Table 1, Tables 3, 4, the performance of the four models has a decline in this scenario. The Dice score decreases by 0.015, 0.026, 0.030, and 0.011 for UNet, ConvLSTM, Echo-LSTM and Echo-ODE, respectively. Our proposed Echo-ODE achieves the least decrease and highest mean segmentation Dice score. It also performs the best in terms of temporal consistency and phase detection error.

**TABLE 5:** Comparison of segmentation performance of patients with arrhythmia. ↑ : higher values indicate better performance. ↓ : lower values indicate better performance. The best results are highlighted in bold.

Method	Region similarity (↑)		TC (↓)	Phase detection (↓)		
	IoU	Dice		ED	ES	Overall
UNet	0.718	0.817	0.401	2.24	2.38	2.30
ConvLSTM	0.696	0.805	0.400	**1.57**	2.29	1.93
Echo-LSTM	0.689	0.794	0.415	1.93	**1.94**	1.94
Echo-ODE	**0.721**	**0.823**	**0.358**	1.58	1.98	**1.78**

#### 3.2.6 Other tasks

**FIGURE 5:**
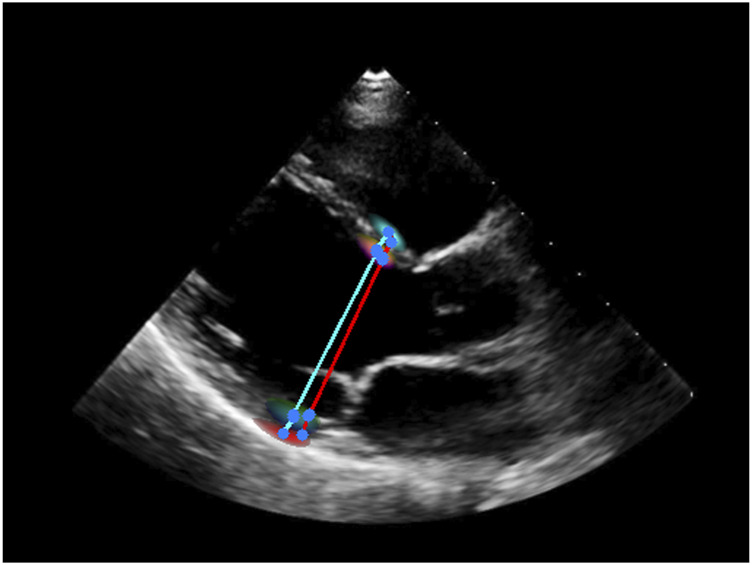
An example of measurement of ventricular dimensions. Red: label. Cyan: prediction. Blue: start/end point of the line.

To test the efficiency of Echo-ODE in accomplishing other tasks, we replace the segmentation target decoder with a measurement decoder to measure the ventricular dimensions. The measurement decoder is similar to the original target decoder, but the output head predicts the probability heatmaps for the beginning and ending points of IVS thickness, LVID dimension, and LVPW thickness respectively, which is the $T_{S}$ in this scenario. The center of gravity of the output heatmaps is regarded as the points. At inference time, low-confidence pixels (with a score less than 0.3) are ignored when calculating the centroid. Ventricular dimensions are calculated using these points and the image resolution. Echo-ODE achieves an MAE of 1.0 mm (95 % CI, 0.9–1.1 mm) for IVS thickness, 2.4 mm (95 % CI, 2.1–2.7 mm) for LVID, and 1.4 mm (95 % CI, 1.2–1.6 mm) for LVPW thickness, which shows very close performance to Duffy et al. (2022), where the MAEs are 1.2 mm, 2.4 mm, and 1.4 mm, respectively. These results demonstrate that Echo-ODE can perform the measurement task. An example in which we overlay the heatmaps on the echocardiography is displayed in Figure 5. The ground truth and predicted measurement lines are highlighted in red and cyan, respectively. As shown, the heatmaps are distributed along the boundaries and the annotated points also lie within the range of the heatmap with high confidence.

### 3.3 Performance on public dataset

#### 3.3.1 EchoNet-dynamic

The experiments are also carried out on the public EchoNet-Dynamic dataset with our model. We compare the results with the reference of Ouyang et al. (2020) who published the dataset, and another reference MAEF-Net by Zeng et al. (2023) who also conducted the tasks of segmentation and phase detection. Ouyang et al. perform an image-level semantic segmentation using the Deeplabv3 architecture. MAEF-Net uses a multi-attention mechanism to guide the network in capturing heartbeat features effectively and incorporates a deep supervision mechanism and spatial pyramid feature fusion to enhance feature extraction capabilities. The results are shown in Table 6.

**TABLE 6:** Comparison of results on public dataset EchoNet-Dynamic. ↑ : higher values indicate better performance. ↓ : lower values indicate better performance. The best results are highlighted in bold.

References	Dice (↑)			TC (↓)	Phase detection (↓)		
	ED	ES	Overall		ED	ES	Overall
Ouyang et al. (2020)	0.927	0.903	-	-	-	-	-
Zeng et al. (2023)	**0.939**	**0.917**	**0.931**	-	2.28	2.43	2.36
Echo-ODE (Ours)	0.934	0.915	0.927	0.209	**2.00**	**1.95**	**1.97**

We achieve better performance in dice similarity of segmentation than Ouyang et al., but slightly worse than Zeng. MAEF-Net uses a multi-attention mechanism to gather spatial and temporal information, which provides more low-level semantic features. As discussed earlier, Echo-ODE loses many low-level features, so the dice coefficient of segmentation struggles to surpass advanced 2D models. However, Echo-ODE performs better in the phase detection task. We can draw a preliminary conclusion that the prediction of Echo-ODE is more temporally stable and continuous, even though Zeng et al. do not report the temporal consistency of their segmentation.

#### 3.3.2 CAMUS

**FIGURE 6:**
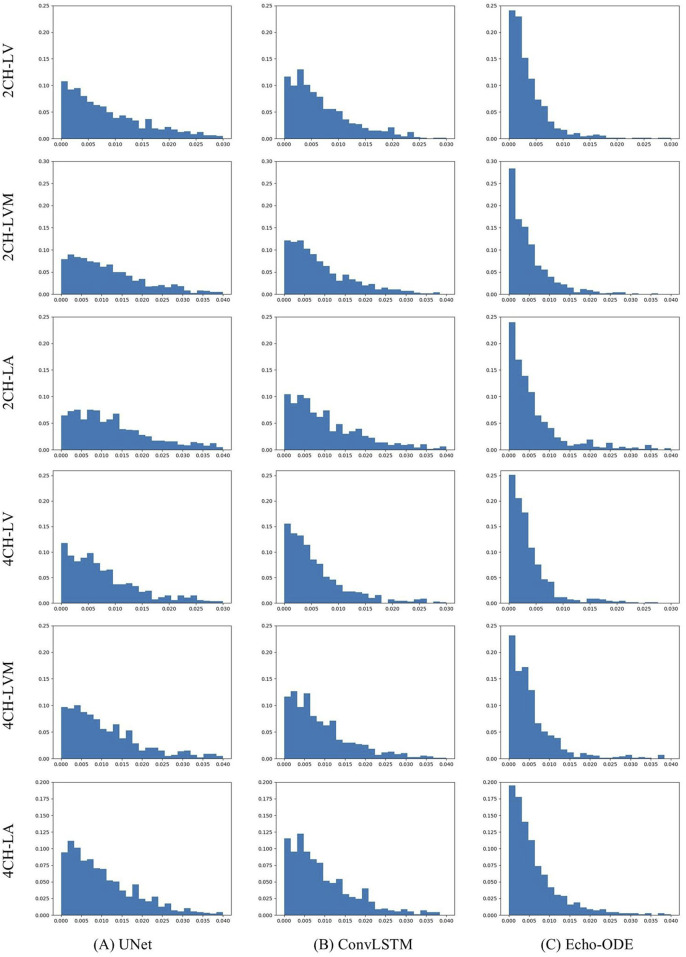
Distributions of $TC_{D}$ by **(A)** UNet, **(B)** ConvLSTM, **(C)** Echo-ODE.

Since CAMUS is fully annotated, we can evaluate performance not only on ED and ES frames, but also compare the temporal consistency of the predicted video segmentation with the ground truth. To this end, we conduct experiments on CAMUS. During training, only annotations of ED and ES frames are used, and testing is applied to the entire video. The results are summarized in Table 7, and the distributions of $TC_{D}$ for the three methods and the three structures are illustrated in Figure 6.

**TABLE 7:** Comparison of performance on public dataset CAMUS. ↑ : higher values indicate better performance. ↓ : lower values indicate better performance. The best results are highlighted in bold.

View	Method	Dice (↑)				TC (↓)	$TC_{D}$ ( ↓ )		
		LV	LVM	LA	Mean		LV	LVM	LA
2CH	UNet	0.934	0.882	0.893	0.903	0.1658	0.010	0.013	0.019
	ConvLSTM	0.936	0.877	0.885	0.899	0.176	0.007	0.010	0.016
	Echo-ODE	**0.939**	**0.889**	**0.893**	**0.907**	**0.1175**	**0.004**	**0.006**	**0.009**
4CH	UNet	**0.943**	**0.880**	**0.912**	**0.911**	0.1612	0.009	0.012	0.012
	ConvLSTM	0.934	0.866	0.904	0.901	0.1543	0.007	0.010	0.013
	Echo-ODE	0.938	0.875	0.893	0.902	**0.1199**	**0.005**	**0.007**	**0.009**

As shown, the three methods demonstrate comparable overall accuracy, but Echo-ODE has much smaller TC and $TC_{D}$ than the other two methods. It is worth noting that the TC of the ground truth is 0.0812 and 0.0911 for the 2CH and 4CH views, respectively. The TC of Echo-ODE is very close to the ground truth, and the distributions of $TC_{D}$ by Echo-ODE are more concentrated near zero and have fewer significantly large errors. Clearly, Echo-ODE shows the best performance in temporal consistency.

### 3.4 Ablation study

As described in Section 2.4, we utilize a reconstruction block (Rec) on the baseline to enhance the dynamic representation of our model and add the skip connections (Skip) from the reconstruction path to the segmentation path. To show the effectiveness of these two blocks, we compare the results of these different methods and the results are listed in Table 8. Clearly, both Rec and Skip contribute to improvements in segmentation accuracy, but share similar temporal stability. This further confirms that the NODE framework has the property of producing temporally consistent predictions, regardless of how the decoders are designed.

**TABLE 8:** Results of the ablation study. ↑ : higher values indicate better performance. ↓ : lower values indicate better performance. The best results are highlighted in bold.

Method	Dice (↑)							TC (↓)	Phase detection (↓)		
	LA	IVS	LVPW	LV	RV	RVAW	Mean		ED	ES	Overall
Baseline	0.893	0.865	0.744	0.920	0.917	0.589	0.821	0.3090	**1.32**	2.08	1.75
Baseline + Rec	0.903	0.863	0.745	0.920	0.920	0.593	0.824	0.3063	1.45	**1.92**	1.71
Baseline + Rec + Skip	**0.907**	**0.874**	**0.745**	**0.922**	**0.931**	**0.622**	**0.834**	**0.2832**	1.41	1.94	**1.70**

### 3.5 Computational complexity

We compare the computational complexity among different models. The number of floating-point operations ( # FLOPs) and the number of parameters ( # Params) are obtained using the function thop.profile() in Python . In addition, we provide runtime analysis in our private test dataset. The results are summarized in Table 9. UNet is an image-level model, so the # FLOPs and # Params are smaller than those of the other three methods. Echo-LSTM has an extra reconstruction branch compared to ConvLSTM, so it has higher computational complexity. The only difference between Echo-ODE and Echo-LSTM is the bottleneck, so, as shown in the table, the # FLOPs of Echo-ODE are 1.19B greater than those of Echo-LSTM in our experimental setting. Since we use shared parameters in temporal modeling, the difference in # Params among the three video-level models is not particularly large. Due to the fewer parameters and single-frame input, UNet requires significantly less training time than other models. In Echo-ODE, the optimization of ODE blocks is more time consuming, resulting in a training time approximately twice that of LSTM-related models. The same conclusion can be drawn in terms of the inference time of single forward propagation (referring solely to the time of data flow through the model, excluding data reading and other precesses). It should be noted that a single forward propagation can produce 1, 4, 4, and 7 frames of segmentation for UNet, ConvLSTM, Echo-LSTM, and Echo-ODE respectively in our experimental setups. Thus, the time-consuming differences for video segmentation across these four models are not very large (1.22s, 1.26s, 1.56 and 1.95s respectively). The mean video length in the test dataset is 158. Therefore, although Echo-ODE has a larger model size and requires more training time, the inference time difference for long video segmentation is not significant (within 2 seconds). If the video is further sparsely sampled and input into similar trained Echo-ODE models, the prediction time for video segmentation will be further reduced. Considering the potential benefit of more temporally consistent video segmentation, it is worth the extra time.

**TABLE 9:** Computational complexity of different methods.

Method	# FLOPs	# Params	Training time (per epoch)	Inference time (single forward)	Inference time (per video)
UNet	0.76B	4.3M	49.5s	0.3 ms	1.22s
ConvLSTM	5.77B	7.1M	185.5s	3.1 ms	1.26s
Echo-LSTM	7.44B	7.6M	237.5s	3.6 ms	1.56s
Echo-ODE	8.63B	8.7M	469.6s	9.1 ms	1.95s

## 4 Discussion

Segmentation of video echocardiograms plays an essential role in clinical diagnosis. Accurate and temporally consistent segmentation contributes to the reliable detection of ED and ES frames, quantification of cardiac functions, and other tasks. In this work, we propose a new framework, Echo-ODE, in which the heart is regarded as a dynamical system. We model the representation of dynamics using NODE. Echo-ODE learns the spatio-temporal relationships of the input video and outputs dense and continuous predictions. The main contributions of this paper are: (1) We propose Echo-ODE, an innovative framework that models the video echocardiogram as a dynamical system using NODE. The output of our model is dense and continuous. To our knowledge, this is the first work to apply NODE to echocardiography. (2) We achieve whole-heart video segmentation of the parasternal long-axis view (PLAX), including six cardiac structures, using our private dataset. (3) We introduce a new temporal consistency metric. (4) Experiments on both private and two public datasets demonstrate that Echo-ODE’s video segmentation is more temporally stable and consistent, which significantly contributes to improved performance in downstream tasks. Echo-ODE shows great potential to perform reliable and fully automatic video echocardiogram analysis.

The experimental results verify that Echo-ODE can learn the dynamic representation of the heart from video reconstruction and interpolation (Figure 2). Table 1 shows that Echo-ODE has a segmentation accuracy comparable to that of UNet and ConvLSTM. Since our model directly decodes the output of NODE without skip connections from the encoder path, it loses much low-level semantic information. Under this premise, it is encouraging to achieve comparable accuracy with the other methods. Echo-ODE performs better than Echo-LSTM, indicating that NODE is more suitable for modeling cardiac dynamic information than LSTM in our scenario. Additionally, a better segmentation accuracy for RVAW demonstrates that Echo-ODE has better video understanding and can more effectively deduce missing structures.

Table 3 shows that Echo-ODE video segmentation has the best temporal consistency in all cardiac structures. This advantage arises from the continuous dynamic representation of NODE, which contributes to the automatic analysis of downstream tasks. For example, the MAE of phase detection using LV segmentation by Echo-ODE is the smallest (Table 4).

Table 5 shows that although all models perform slightly worse in dealing with patients with arrhythmias, Echo-ODE is the least affected and achieves the best segmentation accuracy. UNet is the second least affected because it is an image-level model with minimal temporal correlation. Segmentation accuracy for LSTM-based methods experiences the largest decline due to their discrete modeling of video frames, which does not adapt well to temporal irregularity. Furthermore, when we change the decoder branch to the task of key-point detection for cardiac measurement, Echo-ODE performs well. Experiments on two public datasets, including views of 4CH and 2CH, also lead to similar conclusions (Tables 6, 7), demonstrating that our model can be applied to various sectional views. These experiments highlight the robustness of our proposed model in handling various tasks and cardiac conditions in clinical scenarios. The ablation study (Table 8) shows that both the reconstruction block and the skip connection block in our model contribute to better performance.

Table 9 shows the computational complexity of the four models. Echo-ODE involves temporally continuous calculations, so its #FLOPs are higher than the other three methods. However, the #Params are not significantly larger because all these video-level methods use shared parameters in temporal modeling. Although Echo-ODE requires more time for a single forward propagation, it can predict nearly twice as many frames of segmentation. Considering the dense output scheme of the differential equation solver, Echo-ODE enables more continuous predictions without additional computational resources or time, making it more computationally efficient, so it is worth the extra time on the premise of its real-time prediction.

Our proposed Echo-ODE has several natural advantages. Firstly, it is based on the biodynamical modeling of specific scenarios, as explained in the introduction chapter. This not only strengthens the modeling ability, but also enhances the interpretability of the network. It offers a more functional alternative to optical flow. Secondly, the input of Echo-ODE does not need to have a constant time duration. It can better adapt to data collected from multicenter and multimachine setups, making it more suitable for transfer learning or multicenter learning. Last but not least, the continuity of the output can be utilized for super-resolution in the time dimension. For example, applying Echo-ODE to M-mode echocardiography can produce the corresponding vector graph and contribute to more accurate diagnoses.

However, this work has limitations. Our model and experiments leave much room for further exploration. (1) We need to gain a deeper insight into the hidden space and the derivative function to explore the interpretability of the model, and how are they connected to the biomechanics or underlying dynamical system. (2) Finding a way to reduce the loss of low-level semantic information is crucial. Although our reconstruction block strengthens the learning of dynamic representation, it cannot yet compensate for the loss of detailed information. (3) The computational cost of Echo-ODE is relatively higher than that of other mainstream CNN models. (4) We seek a more explainable method to integrate overall dynamic information into the neural derivative function. (5) The core experimental comparison in this work is deliberately focused on Echo-ODE (continuous modeling) against the most direct and conceptually relevant RNN models (discrete modeling). Providing a broader performance comparison across different methodological families is a planned focus of our immediate future work (6) The generalizability of Echo-ODE on more datasets, other echocardiographic views, patient cohorts and clinical relevance should be further validated. (7) We can explore the impact of various ODE solvers on the performance of this model. (8) The parameter settings of Savitzky-Golay filter used in the phase detection task is rigid. Considering that it may be dependent on factors such as recording frame rates or fibrillation, future work will focus on dynamically adapting filter parameters for a more generalizable implementation.

## 5 Conclusion

In this work, we propose Echo-ODE, an NODE-based network that learns the biodynamics of video echocardiograms. It achieves promising performance on both private and public datasets. The results demonstrate that the temporal stability and consistency of the predictions by Echo-ODE have great potential to enable reliable, fully automatic echocardiogram analysis.

## Data Availability

The public data used in this study is available in the article. The private data are not publicly available because of privacy and security concerns, but are available upon reasonable request from the corresponding author.
