# Supplementary material for: Echo-ODE: A dynamics modeling network with neural ODE for temporally consistent segmentation of video echocardiograms
Source: Front Physiol. 2025 Aug 18;16:1629121. doi: 10.3389/fphys.2025.1629121 (PMC12399644; doi:10.3389/fphys.2025.1629121)
Supplement: Supplementary file 1 [file DataSheet1.pdf]

Supplementary Material

1 SUPPLEMENTARY FIGURES

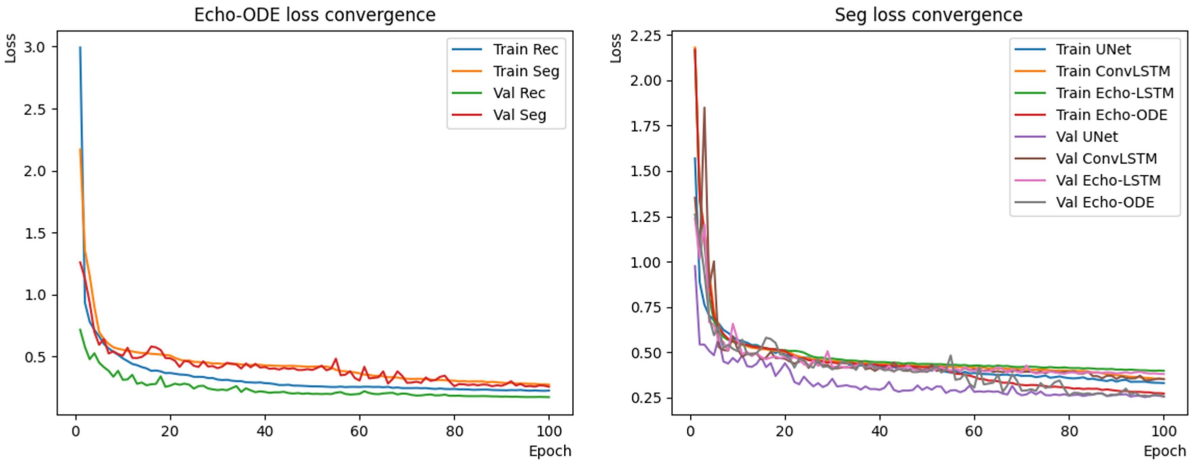

(A) (B)

Figure S1. Loss convergence behavior of the training process. Rec: reconstruction loss. Seg: segmentation loss. (A) The training and validation loss of Echo-ODE. The reconstruction loss depicted in the figure has been scaled by the weighting coefficient λ. (B) The segmentation loss compared among the four methods (UNet and ConvLSTM have no reconstruction loss, so only segmentation loss is compared).
